# SGLT2 Inhibitors, What the Emergency Physician Needs to Know: A Narrative Review

**DOI:** 10.3390/jcm10092036

**Published:** 2021-05-10

**Authors:** Henri Lu, Hortense Lu, Christophe Kosinski, Anne Wojtusciszyn, Anne Zanchi, Pierre-Nicolas Carron, Martin Müller, Philippe Meyer, Jehan Martin, Olivier Muller, Roger Hullin

**Affiliations:** 1 Service of Cardiology, Cardiovascular Department, Lausanne University Hospital, 1011 Lausanne, Switzerland; olivier.muller@CHUV.ch (O.M.); Roger.Hullin@chuv.ch (R.H.); 2 Emergency Department, Saint-Joseph Hospital, 75014 Paris, France; lu.hortense@gmail.com; 3 Service of Endocrinology, Diabetes and Metabolism, Lausanne University Hospital, 1011 Lausanne, Switzerland; Christophe.Kosinski@chuv.ch (C.K.); Anne.Wojtusciszyn@chuv.ch (A.W.); Anne.Zanchi@chuv.ch (A.Z.); 4 Emergency Department, Lausanne University Hospital, 1011 Lausanne, Switzerland; Pierre-Nicolas.Carron@chuv.ch; 5 Emergency Department, Bern University Hospital, 3010 Bern, Switzerland; martin.mueller2@insel.ch; 6 Cardiology Service, Geneva University Hospitals, 1205 Geneva, Switzerland; Philippe.meyer@hcuge.ch; 7 Emergency Department, Geneva University Hospital, 1205 Geneva, Switzerland; jehan.martin@hcuge.ch

**Keywords:** diabetes, heart failure, renal, guidelines

## Abstract

Canagliflozin, dapagliflozin, empagliflozin, and ertugliflozin belong to a class of antidiabetic treatments referred to as sodium-glucose cotransporter 2 inhibitors (SGLT2 inhibitors, or SGLT2is). SGLT2is are currently indicated in North America and in Europe in type 2 diabetes mellitus, especially in patients with cardiovascular (CV) disease, high CV risk, heart failure, or renal disease. In Europe, dapagliflozin is also approved as an adjunct to insulin in patients with type 1 diabetes mellitus. New data provide evidence for benefits in heart failure with reduced ejection fraction and chronic kidney disease, including in patients without diabetes. The use of SGLT2is is expected to increase, suggesting that a growing number of patients will present to the emergency departments with these drugs. Most common adverse events are easily treatable, including mild genitourinary infections and conditions related to volume depletion. However, attention must be paid to some potentially serious adverse events, such as hypoglycemia (when combined with insulin or insulin secretagogues), lower limb ischemia, and diabetic ketoacidosis. We provide an up-to-date practical guide highlighting important elements on the adverse effects of SGLT2is and their handling in some frequently encountered clinical situations such as acute heart failure and decompensated diabetes.

## 1. Introduction

Sodium glucose-cotransporter 2 inhibitors (SGLT2is) are a class of oral antihyperglycemic agents that block glucose and sodium reabsorption in the proximal tubule of the kidney, causing glucosuria and osmotic diuresis. They improve glycemic control in patients with type 2 diabetes mellitus (T2DM) and provide cardiovascular (CV) and renal benefits independently of diabetes status [1,2,3].

Canagliflozin, dapagliflozin, empagliflozin, and ertugliflozin are indicated as first- or second-line treatments in T2DM individuals with CV disease, high CV risk, heart failure, and chronic kidney disease (CKD) both in Europe and North America, provided that in some European countries, their use may be prohibited if kidney clearance is below 45 mL/min [4,5]. Dapagliflozin is also indicated in patients with heart failure with reduced ejection fraction (HFrEF) independently of diabetes status and, in Europe, as an adjunct to insulin in T1DM [6,7]. Despite an overall favorable safety profile, a few side effects of these medications are important to be aware of, especially in the emergency setting, such as conditions related to volume depletion (hypotension, acute kidney injury), genitourinary infections, and euglycemic diabetic ketoacidosis (eDKA) [8]. As SGLT2is will be administered in a substantial number of patients in the coming years, emergency physicians should be aware of the action of these drugs and their side effects. In this review, we present practical considerations and recommendations for emergency department (ED) physicians, focusing on side effects and the management of SGLT2is in particular clinical situations.

## 2. Adverse Events Related to SGLT2is

Adverse events are categorized as very frequent (incidence of ≥10%), frequent (≥1% and <10%), uncommon (≥0.1% and <1%), rare (≥0.01% and <0.1%) and very rare (<0.01%), using the Council for Organizations of Medical Sciences working group definitions, and available evidence in literature [9]. 

### 2.1. Genitourinary Infections (Frequent to Very Frequent)

An increased risk of genital mycotic infection is associated with SGLT2is, particularly in women (vulvovaginitis) and uncircumcised men (balanitis). Although it is the most common adverse event, most infections are mild or moderate [10]. In a meta-analysis including 36689 patients, canagliflozin, dapagliflozin, and empagliflozin were associated with higher risks of genital mycotic infections compared with placebo, with respective odd ratios (ORs) ranging from 3.64 (95% confidence interval (CI), 2.87–4.63) for empagliflozin to 4.99 (95% CI, 3.74–6.67) for canagliflozin [11].

Concerning the risk of urinary tract infections (UTIs), data are less consistent. Reports of pyelonephritis and complicated UTIs (urosepsis) have prompted the EMA and FDA to add warnings about an increased risk of UTIs [12]. However, in a large meta-analysis which included 110 trials, SGLT2is did not demonstrate an increased risk of UTIs [13]. These data were recently completed by a large population based cohort study which compared SGLT2is with dipeptidyl peptidase-4 (DPP-4) inhibitors and glucagon-like peptide 1 receptor agonists (GLP-1 RAs) and did not show an increased risk of UTIs associated with SGLT2is [14]. In practice, patients taking SGLT2is should be regularly informed about the importance of maintaining good local hygiene and should be educated about the signs and symptoms of genital mycotic infection and UTIs. The management of the latter is not different from usual care, with the administration of local/oral antifungal therapy or oral/intravenous anti-biotherapy as needed.

### 2.2. Hypoglycemia (Frequent)

SGLT2is inherently present a low risk of hypoglycemia because of their insulin-independent pathway of action [8]. In clinical trials, hypoglycemic events were rare and not more frequent in nondiabetic patients [1,2]. However, a concomitant use of insulin or insulin secretagogues (glinides, sulfonylureas) may increase the risk of hypoglycemia. Some authors have therefore suggested reducing the dose of sulfonylurea or glinide by 50% or the basal insulin dose by 20% when starting a SGLT2i, especially when glycated hemoglobin (HbA1C) at baseline is normal or when the patient has a known history of hypoglycemic events [15]. Episodes of hypoglycemia in the ED should be managed according to standard protocol, with administration of oral or intravenous glucose, and temporary withholding of the SGLT2i. The dose of other antihyperglycemic agents should subsequently be adapted.

### 2.3. Volume Depletion and Acute Kidney Injury (Frequent)

Because of its effect on osmotic diuresis, SGLT2is may cause symptomatic hypotension or dehydration (incidence of 1.2% to 1.5%), especially in elderly patients or those already taking diuretics [16]. In patients presenting to the ED with hypotension or symptoms or signs of dehydration, temporary withholding of SGLT2is may be considered.

Regarding the risk of acute kidney injury (AKI), reports have provided conflicting conclusions. In 2016, the FDA issued warnings regarding the use of canagliflozin and dapagliflozin after 101 cases of AKI, some requiring dialysis [17]. Most occurred within 1 month of SGLT2i initiation. This observation has been challenged by a meta-analysis of the main clinical trials, which suggested the risk of AKI may slightly decrease via the reno-protective effects of SGLT2is [18]. In practice, a modest decrease in estimated glomerular filtration rate (eGFR) of around 3–4 mL/min/1.73 m^2^ is expected when starting a SGLT2i [16]. Kidney function should be assessed before treatment initiation and monitored thereafter, especially in patients with comorbidities or medications predisposing to AKI.

In patients presenting to the ED with severe AKI, SGLT2i therapy and other potentially nephrotoxic drugs should be withheld, with the correction of any electrolyte imbalance and the initiation of a standard protocol for fluid resuscitation in severe AKI resulting from circulatory hypovolemia and hemodialysis as a last resort, if needed.

### 2.4. Euglycemic Diabetic Ketoacidosis (Rare)

First reported in 2015 [19], euglycemic diabetic ketoacidosis (eDKA) associated with SGLT2is is a rare and life-threatening condition. On a physio-pathological level, it is thought that the lowered plasma glucose levels induced by glucosuria reduce the amount of insulin while increasing glucagon release [20]. The increased glucagon/insulin ratio is responsible for increased lipolysis and upregulated ketone body production (β-hydroxybutyrate, acetoacetate), which may lead to ketoacidosis [20]. Because of the lowered plasma glucose levels related to glucosuria, ketoacidosis under SGLT2i is generally characterized by the absence of major hyperglycemia (<250 mg/dL), hence the name eDKA. Delays in diagnosis can be important because emergency physicians may not be used to ketoacidosis without severe hyperglycemia.

In clinical trials investigating patients with T2DM and those with HFrEF, SGLT2i-associated DKA was rare, with incidence rates ranging from 0.1% (empagliflozin) to 0.6/1000 patient-years (canagliflozin) [21,22,23]. In fact, a real-world propensity-matched study found that SGLT2is were twice more likely to cause eDKA compared with DPP-4 inhibitors [24]. Using the FDA Adverse Event Reporting System (FAERS), Blau and colleagues estimated that SGLT2is increase the risk of eDKA seven-fold [25]. The reason why these observations are different from the clinical trials is unclear. However, participants in randomized trials receive closer medical attention, and this may have contributed to lowering the risk of eDKA.

EDKA should systematically be considered in patients taking SGLT2is, regardless of blood sugar levels presenting with nausea, vomiting, malaise, or abdominal pain or, in more severe cases, altered consciousness, Kussmaul breathing, or clinical signs of shock [19]. This is especially true when an additional trigger is present such as fasting, dehydration, discontinuation of insulin therapy, surgery, infections, or excessive alcohol intake [26]. EDKA may also be found in patients with T1DM, for example, when the insulin pump is defective, or in pregnant women with T1DM, as pregnancy may cause significant physiological glucosuria. Initial evaluation is similar to classic DKA and should rapidly include screening with serum pH. If the latter is below 7.3, rapid β-hydroxybutyrate capillary checking should be considered [19].

In the ED setting, physicians may be confronted with other forms of acidosis, particularly metformin-associated lactic acidosis (MALA), which should not be confused with eDKA in patients treated by a SGLT2i and metformin at the same time. The difference between eDKA and MALA is the absence of β-hydroxybutyrate elevation in MALA, but the two disorders can coexist in theory, especially in patients with CKD [27]. Diagnostic criteria of eDKA, as defined by the American College of Endocrinology, and MALA, are presented in Table 1.

Once the diagnosis of eDKA is confirmed, SGLT2is should immediately be discontinued, and treatment should be started including fluid resuscitation, insulin with concomitant glucose infusion, careful electrolyte and glycemia monitoring, and treatment of the underlying trigger if feasible [28]. Patients taking SGLT2is should be instructed to maintain sufficient oral hydration and carbohydrate intake and to stop the drug in the case of emergency surgery or unexpected external severe stress events [19].

### 2.5. Necrotizing Fasciitis of the Perineum (Very Rare)

Necrotizing fasciitis of the perineum, also known as Fournier’s gangrene (FG), is a potentially fatal acute necrotic infection of the subcutaneous tissues around the genital or perianal regions. Classic risk factors for FG include hypertension, obesity, tobacco use, immunosuppression, heart failure, and T2DM [29]. Using the FAERS, Bersoff-Matcha and colleagues identified 55 cases of FG in patients receiving SGLT2is from March 2013 to January 2019 versus 19 cases in patients receiving other classes of antidiabetic therapy during a 35-year period in the U.S. population [30]. The physio-pathological explanation behind this observation is unknown.

FG should be suspected in any patient taking a SGLT2i who presents with fever along with pain, erythema, or swelling of the genital or perianal area. If diagnosed, immediate discontinuation of the SGLT2i, hospitalization, close monitoring, prompt introduction of broad-spectrum anti-biotherapy, and surgical debridement, if necessary, are required [10].

### 2.6. Fractures (Unknown Incidence)

Canagliflozin was the only SGLT2i associated with a slightly higher risk of fractures, most of them nonvertebral, in the CANVAS-Program trial, but this has not been confirmed in subsequent studies [21]. Possible explanations include a greater risk of falls due to volume depletion and a reduction in bone density [31].

### 2.7. Lower Limb Amputations (Unknown Incidence)

An increased risk of lower limb amputations (LLA) with canagliflozin, mostly toe and metatarsal ones, was reported in the CANVAS-Program trial with a HR of 1.97 (95% CI 1.41–2.75) [21]. Although dapagliflozin and empagliflozin were not associated with the same risk in their respective clinical trials [22,23], a meta-analysis confirmed the slight increase in the risk of LLA for SGLT2is compared to controls or placebo. The mechanism by which SGLT2is might increase the risk of amputations is unknown, and whether it concerns all drugs remains controversial.

SGLT2is should be used with caution in patients with previous amputations, active cutaneous ulcers, or lower extremity artery disease [16]. In the case that peripheral arterial disease is suspected, screening should be rapidly performed.

### 2.8. Stroke

Of note, some data have reported an increased rate of stroke associated with SGLT2is, the mechanism of which is not fully understood [32]. This was, however, not confirmed in a large meta-analysis, which did not find any significant differences between dapagliflozin, canagliflozin, and empagliflozin compared with control groups [33].

Table 2 summarizes adverse events, their respective incidence rates, and proposed management.

## 3. Use of SGLT2is in Particular Clinical Situations

In general, it is advised to discontinue SGLT2is in patients undergoing urgent surgery or hospitalized with any acute serious medical condition (e.g., infections, stroke, acute kidney or liver dysfunction) because of the risk of eDKA. In case of scheduled surgery, the FDA recommends discontinuing SGLT2is 72 h prior to intervention (96 h for ertugliflozin) [34].

However, in certain acute clinical situations, discontinuing the SGLT2i may be debatable. In the following section, we discuss the management of patients taking SGLT2is at baseline, presenting to the ED in four different clinical situations.

### 3.1. Acute Heart Failure

It is estimated that more than 26 million people suffer from heart failure worldwide, with up to 77% presenting at least once to the ED with decompensated acute heart failure (AHF) [35]. As such, the ED serves as the portal of entry for the majority of AHF admissions, and managing SGLT2is in patients presenting with AHF is already and will become an increasingly frequent challenge in the coming years. The role and use of SGLT2is in AHF has not yet been investigated in large-scale trials. However, in a randomized pilot study comparing empagliflozin against placebo in 80 patients with AHF (with and without T2DM), Damman et al. showed that empagliflozin was associated with a greater urinary output and a reduction in a combined outcome of worsening HF, death, and/or rehospitalization for AHF at 60 days [36]. In a position statement from the Heart Failure Association of the European Society of Cardiology, SGLT2is are considered as third-line diuretic treatment with added loop diuretics, after thiazides or acetazolamide/amiloride, based on their natriuretic and osmotic diuretic effect [37]. In view of these data, in an AHF patient with a SGLT2i at baseline, it seems reasonable to continue the treatment provided the patient does not have any sign of hemodynamic instability and does not present contraindications or side effects as described above (e.g., eDKA, AKI, hypotension). In patients with “wet” (congestive) AHF, temporarily increasing the dose of the SGLT2i (if possible) may be an option if additional diuretic effect is necessary after the initiation of standard diuretic therapy. In patients with “dry” AHF, keeping the same dose of SGLT2i is an option.

### 3.2. Atrial Fibrillation with Rapid Ventricular Response

Literature regarding the management of SGLT2is in the setting of patients presenting with atrial fibrillation (AF) with rapid ventricular response is very scarce. However, in authors’ opinion, the decision to discontinue or not the SGLT2i primarily depends on the hemodynamic tolerance of the AF and the volume status. In patients with hemodynamic instability (symptomatic hypotension, cardiogenic shock) and in those presenting with hypovolemia based on clinical and biological evaluation, it seems reasonable to withhold the SGLT2i in addition to general AF management (initiation of rhythm or rate control, fluid resuscitation, and initiation of anticoagulation, if needed).

### 3.3. Acute Diabetes Decompensation

Acute diabetes decompensation is another commonly encountered condition in the ED and includes hyperosmolar hyperglycemic state (HHS), eDKA, and marked hyperglycemia without hyperosmolar or ketosis conditions. Although historically considered as two distinct clinical entities, HHS and eDKA share the same basic pathophysiologic mechanisms: Significant insulin deficiency and increased concentration of counterregulatory hormones such as glucagon, catecholamines, cortisol, and growth hormone. Furthermore, similar to patients with eDKA, those with HHS frequently may present with signs of dehydration, dry mucous membranes and poor skin turgor, or hypotension [38]. Although no clear recommendation regarding HHS and the use of SGLT2is exists, in patients with a SGLT2i at baseline presenting to the ED with HHS, it seems reasonable to initiate standard HHS treatment: fluid resuscitation, insulin infusion, careful electrolyte and glycemia monitoring, and treatment of the underlying cause.

### 3.4. Gout Attack

SGLT2is have been shown to consistently reduce uric acid concentrations via increased urinary uric acid excretion by approximately 35 45 μmol/L to 45 μmol/L (0.60–0.75 mg/dL). The lowering of uric acid by SGLT2is may partly explain the beneficial CV and renal effects associated with this class of treatments [8]. When compared with traditional hypouricemic treatments (xanthine oxidase inhibitors), their mode of action is different and potentially complementary [39]. Whether SGLT2is may be useful in the management of hyperuricemia is still unknown, but it seems reasonable, in patients with a SGLT2i at baseline and presenting with a gout attack, to continue the SGLT2i, providing they do not have any condition which might put them at risk of developing eDKA (AKI, significant inflammatory syndrome…).

Table 3 summarizes the management of SGLT2is associated with the four precited clinical situations.

### 3.5. Pregnancy

No well-controlled studies of SGLT2is have been performed in the context of pregnancy. According to animal studies, these drugs may affect renal development. Therefore, SGLT2is should be used in pregnant women only if the benefits justify the risk to the fetus [40].

## 4. Conclusions

SGLT2is are antidiabetic drugs for which indications are currently rapidly expanding, as new data show beneficial effects not only in patients with T2DM, but also in those with HFrEF and CKD, regardless of the diabetes status. Overall, they are well-tolerated treatments, with mild genital mycotic infections and volume depletion being the most common adverse events. However, as the number of patients taking SGLT2is increases, emergency physicians may be faced with rarer adverse events, some of which, if left unrecognized, could be life-threatening. The risk of adverse events may be reduced by careful patient information and education about self-monitoring. Although it is advised to discontinue SGLT2is in patients undergoing urgent surgery or hospitalized with any acute serious medical condition, in certain acute clinical situations, such as acute heart failure, this may be debatable, as SGLT2 inhibition may actually yield beneficial effects.

## Figures and Tables

**Table 1:** Diagnostic criteria of euglycemic diabetic ketoacidosis and metformin-associated lactic acidosis [19,27].

Parameters	Laboratory Values	
	**eDKA**	**MALA**
Arterial pH	<7.3	<7.35
β-hydroxybutyrate	≥31 mg/dL (3.0 mmol/L) in children ≥40 mg/dL (3.8 mmol/L) in adults	Normal
Serum ketone	Positive	Negative
Serum lactate	Normal or slightly elevated	>5 mmol/L
Anion gap	>10 mmol/L	>10 mmol/L

eDKA: Euglycemic diabetic ketoacidosis, MALA: Metformin-associated lactic acidosis.

**Table 2:** Adverse events associated with SGLT2is and proposed courses of action.

Adverse Events	Incidence	Practical Considerations
Mycotic genital infections	Very frequent Male C: 34.9 p/1000 p-y E: 5% Female C: 68.8 p/1000 p-y E: 10%	- Local or oral antifungal therapy - Reinforce patient education: Adequate hydration, good local hygiene, and self-monitoring
Urinary tract infections	C: 40 p/1000 p-y D: 1.5% E: 1.8%	- Oral or intravenous anti-biotherapy - Reinforce patient education: Adequate hydration, good local hygiene, and self-monitoring
Hypoglycemia	Frequent C: 50 p/1000 p-y D: 0.7% E: 1.3%	- Administration of oral or intravenous glucose - Withholding of SGLT2i - Hospitalization if required - When restarting antidiabetic therapy, the dose of other antihyperglycemic agents should be adapted: Sulfonylurea or glinide by at least 50% and basal insulin by at least 20%
Hypotension	Frequent C: 26 p/1000 p-y E: 5.1%	- Oral or intravenous fluid administration - Hospitalization if required - Adapt the dose of antihypertensive treatments - Inform patients to maintain adequate oral hydration - Treat concomitant conditions (diarrhea…) - If severe condition related to volume depletion, consider temporary withholding of SGLT2i
Acute kidney injury	Frequent C: 3 p/1000 p-y D: 1.5% E: 1.0%	- Oral or intravenous fluid administration - Hospitalization if required - Adapt the dose of other medications that may cause AKI (nonsteroidal anti-inflammatory drugs, diuretics, renin-angiotensin-aldosterone inhibitors) - Inform patients to maintain adequate oral hydration - Treat concomitant conditions such as diarrhea, if indicated - If severe AKI, consider temporary withholding of SGLT2i
Diabetic ketoacidosis	Rare C: 0.6 p/1000 p-y D: 0.3% E: 0.1%	- Hospitalization in intensive care unit - SGLT2i discontinuation - Fluid resuscitation, intravenous insulin and glucose continuous infusion, careful electrolyte and glycemia monitoring (target: 8-11 mmol/L) - Treatment of the underlying trigger - Inform patients to avoid precipitating factors: Fasting, dehydration, discontinuation of insulin therapy, surgery, infections, or excessive alcohol intake
Fournier’s gangrene	Very rare C, D and E: <0.1%	- Hospitalization and close monitoring - Broad-spectrum anti-biotherapy and surgical debridement, if necessary
Fractures	C:15.4 p/1000 p-y	- Conservative or surgical management - Instruct patients to maintain adequate calcium uptake - Consider osteoporosis screening
Lower limb amputations	C: 6.4 p/1000 p-y D: 1.4%	- Use SGLT2is with caution in patients with previous amputations or peripheral artery disease - Low threshold to screen for peripheral arterial disease - Remind patients to perform regular foot exams

AKI: Acute kidney injury. Incidence rates of side effects are based on the CANVAS-Program [21], DECLARE-TIMI 58 [22], and EMPAREG-OUTCOME [23] trials. They are expressed as number of patients for 1000 patient-years (p/P-y=patients/1000 patient-years) for canagliflozin (C), as percentages for dapagliflozin (D) and empagliflozin (E). SLGT2i: Sodium-glucose cotransporter 2 inhibitor.

**Table 3:** Proposed management of patients taking SGLT2is at baseline in the case of acute heart failure, atrial fibrillation with rapid ventricular response, acute diabetes decompensation, and gout attack.

Conditions	Proposed Course of Actions
Any planned or unplanned surgery Any acute serious medical condition with need of hospitalization, apart from the ones discussed below	Discontinue SGLT2i
Acute heart failure	Hemodynamically stable patients - “Wet” AHF: Continue SGLT2i at the same or at an increased dosage, along with standard diuretic therapy - “Dry” AHF: Continue SGLT2i at the same dosage Hemodynamically unstable patients - Discontinue SGLT2i
Atrial fibrillation with rapid ventricular response	Hemodynamically unstable patients - Discontinue SGLT2i Hemodynamically stable patients - Hypovolemic patients: Discontinue SGLT2i - Euvolemic or hypervolemic patients: Continue SGLT2i at the same dosage
Acute diabetes decompensation	Thoroughly evaluate the patient for eDKA or HHS In case of eDKA or HHS - Immediately discontinue SGLT2i - Associated measures: fluid resuscitation, insulin infusion, careful electrolyte and glycemia monitoring, treatment of the underlying trigger In case of isolated hyperglycemia - Switching to insulin therapy may be preferable
Gout attack	SGLT2i may be continued

AHF: Acute heart failure, eDKA: Euglycemic diabetic ketoacidosis, HHS: Hyperosmolar hyperglycemic state, SLGT2i: Sodium-glucose cotransporter 2 inhibitor.
